# Pterygium surgery combined with the removal of a missed occult iris foreign body detected incidentally during pterygium examination: a case report

**DOI:** 10.1186/s12886-018-1020-y

**Published:** 2019-01-07

**Authors:** Zhitao Su, Houfa Yin, Panpan Ye, Xiaodan Huang, Li Zhang, Jianyun Fu, Xin Xie

**Affiliations:** 1 Eye Center, Second Affiliated Hospital, School of Medicine, Zhejiang University, No. 88 Jiefang Rd, Hangzhou, 310009 China; 2 The Second People’s Hospital of Quzhou, Quzhou, China

**Keywords:** Occult iris foreign body, Combined surgery, Pterygium, Case report

## Abstract

**Background:**

An occult foreign body may be retained in patient with small self-sealing wound and no decreased visual acuity without complete examination. Here we report a case of a retained occult ferrous iris foreign body detected incidentally during pterygium examination.

**Case presentation:**

A 69-year-old man presented to our ophthalmology department because of foreign body sensation and persistent redness in both eyes for 2 years. In the left eye, a pterygium, paracentral corneal opacity and a vertically oval pupil were observed. Ultrasound biomicroscopy and gonioscopy revealed a retained metallic-like foreign body partially embedded in the inferior peripheral iris. Pterygium surgery and the removal of the retained iris foreign body were performed simultaneously. No recurrent pterygium or residual foreign body was found during follow-up.

**Conclusions:**

A thorough history should be obtained and complete physical examination should be performed in patients with ocular self-sealing wounds to prevent missed intraocular foreign bodies, which may result in potential sight-threatening ocular complications.

## Background

Penetrating eye injury is a common condition presenting in emergency services. The frequency of intraocular foreign body (IOFB) following penetrating eye injury is approximately 40% [1–3]. In most cases, IOFBs that could cause severe visual impairment are identified. However, tiny and occult foreign bodies may be missed and retained in patients with small self-sealing wounds and no decreased visual acuity without complete examination [4–6]. The natural progression of a retained IOFB varies depending on its size and location, the toxicity of the material, and risk of infection [7]. A ferrous IOFB can cause deposition of iron molecules in the ocular tissues and result in siderosis bulbi [8, 9]. Serious ocular complications caused by retained IOFBs have been reported [10–13]. In view of sight-threatening complications and the development of better surgical technique, early removal of the retained IOFBs should be performed. Asymptomatic IOFBs have been published [4, 6, 14]. However, to the best of our knowledge, there are no reports of retained iris foreign body without ocular complications. Here we present a case of an occult asymptomatic retained ferrous iris foreign body, which was detected incidentally during pterygium examination.

## Case presentation

**Fig. 1:**
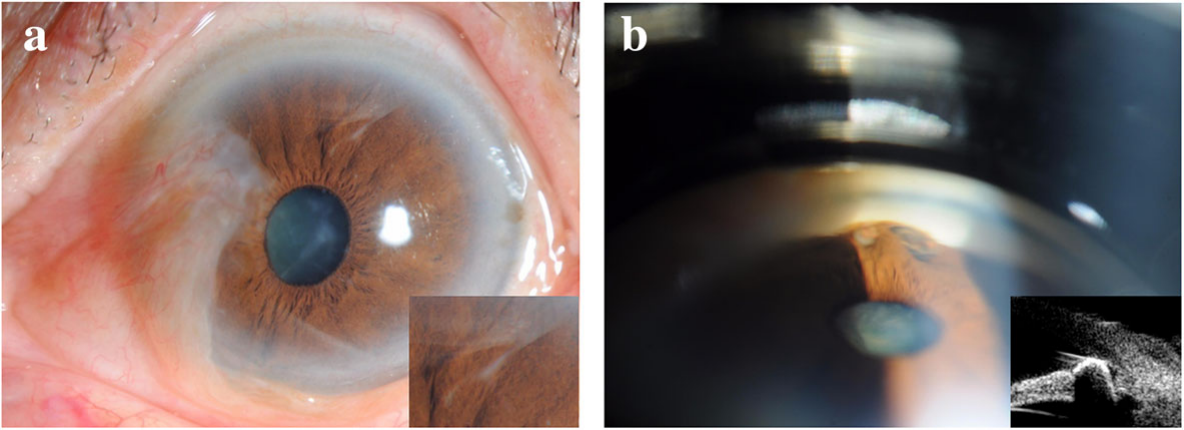
**a** Anterior segment photograph showing pinkish, triangular tissue growth on the nasal cornea, paracentral corneal opacity at 1 o’clock position, and a vertically oval pupil. **b** Gonioscopy and ultrasound biomicroscopy showing a metallic-like foreign body partially embedded in the inferior peripheral iris at 6 o’clock position

All procedures conformed to the Declaration of Helsinki, and written informed consent was obtained from the participant. A 69-year-old man was referred to our ophthalmology department because of foreign body sensation and persistent redness in both eyes for 2 years. The patient suffered a penetrating trauma to the left eye while hammering metal without safety glasses and was treated with topical antibiotics 3 months before. On presentation, the best corrected visual acuity was 20/40 in both eyes, and the intraocular pressure was 14 mmHg in the right eye and 12 mmHg in the left eye. In the left eye, pinkish, triangular tissue growth on the nasal cornea and paracentral corneal opacity at 1 o’clock position were observed. There was no inflammation in the anterior chamber. A vertically oval pupil and a moderate cortical cataract were present (Fig. 1a). Fundus examination revealed no obvious abnormality. Gonioscopy and ultrasound biomicroscopy (UBM) revealed a retained metallic-like foreign body partially embedded in the inferior peripheral iris at 6 o’clock position, which resulted in vertically oval pupil (Fig. 1b). Other foreign bodies were ruled out by B-scan ultrasonography. Except for the pterygium and moderate cortical cataract, the ocular examination was unremarkable in the right eye. Pterygium surgery and removal of the retained iris foreign body were performed simultaneously. After pterygium excision and corneal limbal autograft, a 3.0-mm superonasal clear corneal incision was made using a keratome. The ophthalmic viscoelastic device (Healon) was injected in the anterior chamber through the clear corneal incision to maintain the chamber depth, protect the endothelium and lens integrity, and dislodge the foreign body. The foreign body was captured and removed by a capculorhexis forceps through the clear corneal incision, followed by anterior chamber irrigation to remove the viscoelastic, and hydration of the clear corneal incision. After the surgery, the foreign body, 1.5 mm in width and 2.5 mm in length, was identified as a metallic foreign body by a magnet. Postoperatively, the patient received 0.5% levofloxacin eye drops and 1% prednisolone acetate eye drops 4 times a day for 1 week, and 1% pranoprofen eye drops 4 times a day for 4 weeks. The patient was followed up 1 day, 1 week, 1 month, 6 months, and 12 months after the surgery.

**Fig. 2:**
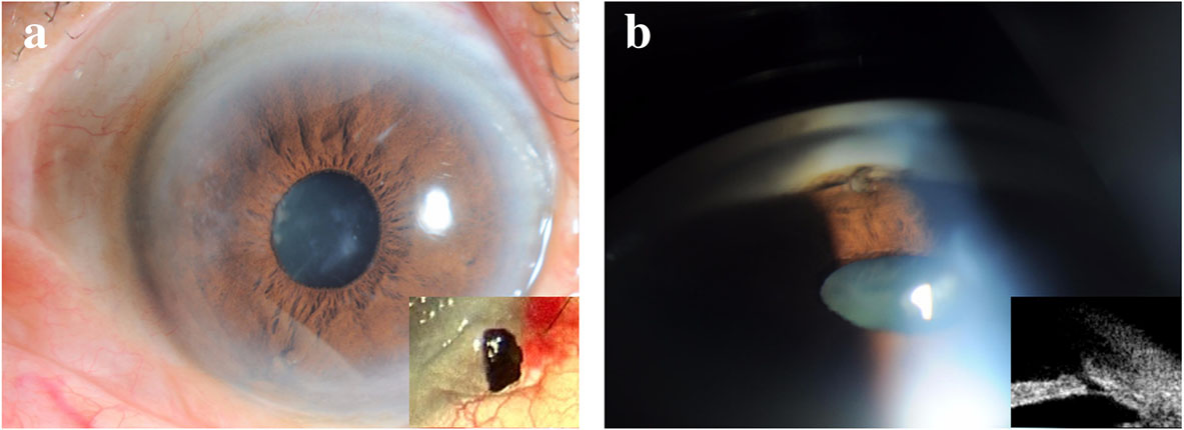
**a** Anterior segment photograph showing a nasal corneal nebula and a round pupil 6 months after the surgery, and removal of the retained iris foreign body during the surgery. **b** Gonioscopy and ultrasound biomicroscopy showing no residual foreign body

Six months postoperatively, the best corrected visual acuity was 20/40 in the left eye, no pinkish tissue growth on the cornea was found, a nasal corneal nebula and a round pupil were observed (Fig. 2a). The moderate cortical cataract and normal fundus were the same as before surgery. No residual foreign body was identified by gonioscopy and UBM (Fig. 2b).

## Discussion and conclusions

Open-globe injury (OGI) can often result in serious visual loss and imposes a significant economic burden on the patient and society. IOFBs account for 18 to 41% of all OGIs [2, 3, 10]. Missed IOFBs in OGI were rare because of the relatively large size of foreign bodies and serious visual loss. However, tiny occult foreign bodies may be missed and retained in patients with small self-sealing wounds and no decreased visual acuity without complete examination [4–6, 14].

Retained iron-containing IOFBs can cause deposition of iron molecules in the ocular tissues, and siderosis bulbi will ensue if the IOFBs are not removed. The clinical findings include iris heterochromia, pupillary mydriasis, cataract formation, secondary glaucoma, and retinal pigmentary degeneration [1, 3, 8, 9]. The length of time that elapsed between the foreign body injury and presentation with siderosis ranged from 3 weeks to 8 years due to the natural course of retained IOFBs [3, 12]. Thus, when detected, an IOFB should be removed promptly to prevent these complications.

Here we report a case of an occult retained ferrous iris foreign body detected occasionally during pterygium examination. The patient suffered a penetrating trauma to the left eye while hammering metal without safety glasses 3 months before. Because of the small self-sealing corneal wound and no decreased visual acuity, he was treated only with topical antibiotics, and the IOFB was missed. Close follow-up should be required in cases of retained ferrous IOFBs without complications [3, 11, 15]. Consideration of impossible close follow-up in this patient and exposure of the ferrous foreign body in the anterior chamber, to prevent potential ocular siderosis and to reduce surgery cost, taking advance of modern surgical technique, pterygium surgery and removal of the retained iris foreign body were performed simultaneously in this case. The foreign body was identified as a metallic foreign body by a magnet after the surgery. Fortunately, the patient in the present case did not exhibit signs of ocular siderosis during follow-up. It may be associated with short duration and less amount of the harmful component.

In some cases, IOFBs (e.g., in the anterior chamber angle, embedded in the iris or lens, or on the pars plicata) cannot be visualized clinically, but can be detected by careful UBM examination [16–18]. Other indications of a possible IOFB include small self-sealing wounds, iris transillumination defect, iris heterochromia, irregular pupil, and focal lens opacities. Computed tomography scan and magnetic resonance imaging are ideal in diagnosing metallic and nonmetallic foreign bodies respectively [15, 19, 20]. However, not all IOFBs can be found even with full examination. A tiny intralenticular foreign body was found during cataract surgery in a patient with negative imaging findings [5]. Therefore, a patient accompanied by penetrating ocular injury should be suspected to have IOFB and should be followed-up closely. When IOFB was detected, it is better to remove the foreign body before irreversible siderosis bulbi occurs.

To the best of our knowledge, this is the first case of an occult missed iris foreign body that was detected incidentally during pterygium examination, followed by pterygium surgery and removal of the retained iris foreign body simultaneously. This case emphasizes the importance of a thorough history and physical examination in patients with ocular self-sealing wounds to prevent missed IOFBs, which may result in potential sight-threatening ocular complications.

## Abbreviations

IOFB: Intraocular foreign body
OGI: Open-globe injury
UBM: Ultrasound biomicroscopy
